# Nematicidal Activity of Cyclopiazonic Acid Derived From *Penicillium commune* Against Root-Knot Nematodes and Optimization of the Culture Fermentation Process

**DOI:** 10.3389/fmicb.2021.726504

**Published:** 2021-11-24

**Authors:** Van Thi Nguyen, Nan Hee Yu, Yookyung Lee, In Min Hwang, Hung Xuan Bui, Jin-Cheol Kim

**Affiliations:** ^1^Department of Agricultural Chemistry, College of Agriculture and Life Sciences, Institute of Environmentally Friendly Agriculture, Chonnam National University, Gwangju, South Korea; ^2^Hygienic Safety and Analysis Center, World Institute of Kimchi, Gwangju, South Korea; ^3^Department of Entomology and Nematology, Gulf Coast Research and Education Center, University of Florida, Wimauma, FL, United States

**Keywords:** cyclopiazonic acid, nematicidal activity, Plackett–Burman, central composite design, response surface methodology, root-knot nematode

## Abstract

Among 200 fungal strains isolated from the soil, only one culture filtrate of *Aspergillus flavus* JCK-4087 showed strong nematicidal activity against *Meloidogyne incognita*. The nematicidal metabolite isolated from the culture filtrate of JCK-4087 was identified as cyclopiazonic acid (CPA). Because JCK-4087 also produced aflatoxins, six strains of *Penicillium commune*, which have been reported to be CPA producers, were obtained from the bank and then tested for their CPA productivity. CPA was isolated from the culture filtrate of *P. commune* KACC 45973. CPA killed the second-stage juveniles of *M. incognita, M. hapla*, and *M. arearia* with EC_50–3 *days*_ 4.50, 18.82, and 60.51 μg mL^–1^, respectively. CPA also significantly inhibited egg hatch of *M. incognita* and *M. hapla* after a total of 28 days of treatment with the concentrations > 25 μg mL^–1^. The enhancement of CPA production by *P. commune* KACC 45973 was explored using an optimized medium based on Plackett–Burman design (PBD) and central composite design (CCD). The highest CPA production (381.48 μg mL^–1^) was obtained from the optimized medium, exhibiting an increase of 7.88 times when compared with that from potato dextrose broth culture. Application of the wettable power-type formulation of the ethyl acetate extract of the culture filtrate of KACC 45973 reduced gall formation and nematode populations in tomato roots and soils under greenhouse conditions. These results suggest that CPA produced by *P. commune* KACC 45973 can be used as either a biochemical nematicide or a lead molecule for developing chemical nematicides to control root-knot nematodes.

## Introduction

Plant-parasitic nematodes (PPNs) are economic burdens in agriculture, owing to their direct and indirect damages that lead to crop yield losses (Bogner et al., 2017); they are estimated to cause an annual yield loss of $173 billion. Root-knot nematodes (RKNs; *Meloidogyne* spp*.)* are the most damaging PPNs to various crops (Termorshuizen et al., 2011; Kim et al., 2016; Gamalero and Glick, 2020). RKNs cause nutrient deficiency, stunting, wilting, chlorosis, reduced tillering, immature fruit drop, and leaf drying (Moens et al., 2009; Palomares-Rius et al., 2017). Among several identified RKN species, *Meloidogyne arenaria*, *M. hapla, M. incognita*, and *M. javanca* are commonly reported worldwide (Anwar and McKenry, 2010; Jones et al., 2013; Dong et al., 2014; Kim et al., 2018; Gamalero and Glick, 2020).

Various chemical nematicides have been used to control RKNs on different crops worldwide. However, most chemical nematicides have a broad spectrum of activity, adversely affecting beneficial soil microbes; they often cause a rapid resurgence of soil-borne pathogens (Sánchez-Moreno et al., 2010; Watson et al., 2017). Therefore, developing new, reduced risk nematicides for RKN control is necessary. Recently, various biological control agents have been studied as alternatives to chemical nematicides to control RKNs. Several bacteria and fungi as biological control agents have been reported to have nematicidal activities against RKNs (Martínez-Medina et al., 2017; Ghahremani et al., 2019). Additionally, numerous nematicidal metabolites from fungal biocontrol agents have been reported for the control of RKNs, including thermolides A and B, omphalotins, ophiobolins, bursaphelocides A and B, illinitone A, speudohalonectriins A and B, dichomitin B, and caryopsomycins A-C (Degenkolb and Vilcinskas, 2016).

Several secondary metabolites isolated from the genera *Penicillium* and *Aspergillus* exhibit antimicrobial, anticancer, antiparasitic, insecticidal, and biocontrol activities (El-Hawary et al., 2020; Toghueo and Boyom, 2020). Furthermore, both these genera are commonly found in the soil and have been known to produce various nematicidal metabolites against RKNs (Siddiqui and Akhtar, 2009; Murslain et al., 2014; Jang et al., 2016). Several studies have reported that *Penicillium commune* has potent inhibitory activity against bacteria such as *Staphylococcus aureus, Pseudomonas fluorescens*, *P. aeruginosa, Bacillus subtilis*, and *Escherichia coli* and fungi such as *Candia glabrata* and *C. albicans* (Gao et al., 2011; Shang et al., 2012; Malhadas et al., 2017). However, the nematicidal metabolites from *P. commune* have not been reported yet.

Optimization of the culture fermentation process is critical to ensure high productivity at a low cost (Calvo et al., 2002; Keller, 2019). The production of secondary microbial metabolites can be enhanced by optimizing physical and chemical conditions (Yang et al., 2016; Zhang et al., 2020). Optimization can be performed using a conventional one-factor-at-a-time approach, a statistical method, or a combination. The conventional approach entails changing one independent factor or variable while keeping the other variables stable. It is labor-intensive, costly, and time-consuming, particularly when many factors are involved. Conversely, the statistical approach is markedly cost-effective, time-efficient, and significantly decreases the number of experimental runs (Rigas et al., 2005; Arul Jose et al., 2013; Nor et al., 2017; Singh et al., 2017; Lim et al., 2019). Three different techniques, such as namely screening, factorial, and response surface methodology, have been used in the statistical method in previous research (Hanrahan and Lu, 2006; Bezerra et al., 2008; No, 2013; Singh et al., 2017; Lim et al., 2020). For screening, the most critical variables affecting maximum response production were identified by the Plackett–Burman design (PBD). Because PBD focuses on selected main effects and disregards the interaction between variables, another step of optimization using a central composite design (CCD) is required. CCD comprises three parts—a factorial portion, central points, and star points—that mathematically evaluate the interactions among various variables and establish the relationship between response and variables (Raissi and Farsani, 2009; El-Naggar et al., 2016; Kundu et al., 2016; Srivastava et al., 2018). Even though several studies on the fermentation process for producing cyclopiazonic acid (CPA) by *Aspergillus flavus* and *P. commune* were conducted in the 1990s using full factorial design, its production concentrations were low (Gqaleni et al., 1996, 1997).

Initially, we screened 200 fungal isolates against *M. incognita* and found that *A. flavus* JCK-4087 showed very strong nematicidal activity. The nematicidal metabolite was identified as CPA through organic solvent extraction, repeated chromatography, and instrumental analysis. However, *A. flavus* JCK-4087 also produced aflatoxins toxic to mammals (Abnet, 2007; Shephard, 2008; Zain, 2011). Therefore, six strains of *P. commune* were obtained from the Korean Agricultural Culture Collection (KACC), Rural Development Administration, Republic of Korea, which are known as CPA producers (Hermansen et al., 1984; Gqaleni et al., 1996; Ostry et al., 2018), were used in this study. Then, one strain was selected for further study. This research was performed to evaluate the potential of CPA as a biochemical nematicide for the control of root-knot nematode diseases. Therefore, the objectives of this study were (1) to isolate and identify CPA from the fermentation filtrate of *A. flavus* JCK-4087 and *P. commune* KACC 45973, (2) to investigate *in vitro* nematicidal activity of CPA against RKNs, (3) to optimize culture conditions using PBD and CCD for CPA production by *P. commune*, and (4) to evaluate the disease control efficacy of ethyl acetate layer extracted from *P. commune* KACC 45973 against root-knot nematode disease in tomato plants.

## Materials and Methods

### Root-Knot Nematode Culture and Preparation

*M. incognita* was obtained from the Korea Research Institute of Chemical Technology (Daejeon, Republic of Korea). Both *M. arenaria* and *M. hapla* were kindly supplied by the National Institute of Agricultural Sciences, Rural Development Administration (Wanju-gun, Jeollabuk-do, Republic of Korea). Second-stage juveniles (J2s) were collected from the populations of *M. arenaria, M. hapla*, and *M*. *incognita* on infected tomato (*Solanum lycopersicum* Mill. cv. Seokwang) plants maintained for at least 2 months at 28 ± 2°C and 75 ± 5% relative humidity (RH) in a greenhouse at Chonnam National University, Korea. The infected tomato plants were uprooted and washed with tap water; the nematode eggs were extracted with 1% sodium hypochlorite (Jang et al., 2016). Egg suspension was passed through a 63 μm sieve and then retained in a 25 μm sieve. The eggs were washed with distilled water and then hatched using the modified Baermann funnel method at 28°C within 5 days (Viglierchio and Schmitt, 1983). Fresh eggs and J2s were used for further experiments.

### Isolation and Identification of Fungal Strain JCK-4087

The method for isolating 200 fungal strains from soil samples collected from the Gwangju campus of Chonnam National University, Sunchang mountain, and Gok-Seong, Korea, was according to Aziz and Zainol (2018). All the isolated fungal strains were cultured on potato dextrose broth medium (PDB; Becton, Dickinson and Company, Sparks, MD, United States) at 25°C with rotary shaking (150 rpm) for 2 weeks and under static conditions for 3 weeks. Each isolated fungal stock was stored at −80°C in 25% glycerol until further use. The nematicidal activity of 200 culture filtrates were tested against second-stage juveniles (J2s) of *M. incognita* as previously described (Cayrol et al., 1989; Nguyen et al., 2018). A fungal strain JCK-4087 was selected based on its high nematicidal activity against *M. incognita* (data not shown).

Total deoxyribonucleic acid (DNA) of JCK-4087 was extracted and amplified in the internal transcribed spacer (ITS) region, and a PCR was performed as previously reported (Nguyen et al., 2019). Amplified fragments were purified and sequenced at Genotech Crop (Daejeon, South Korea). Additionally, β-tubulin (*Bt2*) and calmodulin (*Cmd*) genes were amplified using the primer pair *Bt2a* and *Bt2b* (Glass and Donaldson, 1995) and *Cmd5* and *Cmd6* (Hong et al., 2005), respectively. The result from ITS, *Bbt2*, and *Cmd* sequencing was used to identify JCK-4087 based on the National Center for Biotechnology Information (NCBI) blast database. Multiple sequence alignments were generated with Clustal W and phylogenetic analysis was performed using MEGA version 6 (with the maximum likelihood method), with 1,000 bootstrapping trials (Hasegawa et al., 1985; Tamura et al., 2013; Newman et al., 2016).

### Extraction and Purification of a Nematicidal Metabolite From JCK-4087

The JCK-4087 was cultured on a PDB medium at 25°C on a rotary shaker (150 rpm) for 14 days and then filtered through four cheesecloth layers to segregate culture filtrate and mycelia. Then, the culture filtrate (2.8 L) was partitioned twice with ethyl acetate into a 1:1 ratio (v/v). The crude extract (5.1 g) was loaded onto a chromatography column (3.5 × 60 cm, inner diameter × length) containing silica gel (70–230 mesh, 400 g; Merck, Darmstadt, Germany) and then eluted with chloroform: MeOH (9:1, v/v), yielding eight fractions (F1–F8). These eight fractions were tested for J2s mortality against *M. incognita*. F8 (315 mg), which exhibited nematicidal activity, was further separated using Sep–Pak^®^ Vac 35 cc (10 g) C18 cartridge (Waters Corp., Premier, United Kingdom) with stepwise elution of a mixture of water: methanol (10:0, 9:1, 8:2, 7:3, 6:4, 5:5, 4:6, 3:7, 2:8, 1:9, 0:10, 50 mL per mixture), yielding five fractions (F81–F85). F81 (15.6 mg) showed nematicidal activity against *Meloidogyne* spp. Thus, one nematicidal metabolite (**1**) was purified, and its purity was evaluated via thin-layer chromatography and high-performance liquid chromatography (HPLC) using a Shimadzu LC-20AT HPLC pump and Shimadzu SPD-M20A PDA detector (Shimadzu Corp., Kyoto, Japan) with a C18 column (Xbridge 5 μm, 4.6 × 250 mm, Waters Corp.).

### Structural Determination of a Nematicidal Metabolite

High-resolution electrospray ionization-mass spectrometry (HR-ESI-MS) and nuclear magnetic resonance spectroscopy (NMR) analyses were performed to identify the purified metabolite. HR-ESI-MS analysis of compound **1** was conducted using a Synapt G2 HDMS quadrupole time-of-flight mass spectrometer equipped with an electrospray ion source (Waters Corp.). ^1^H- and ^13^C-NMR, COSY, HSQC, and HMBC were recorded on Bruker Avance III HD 500 MHz instrument (Bruker Biospin GmbH, Rheinstetten, Germany) and dissolved in methanol-d_4_ (Cambridge Isotope Laboratories, Inc., Andover, MA, United States). The internal standard for NMR analysis was tetramethylsilane.

### Quantification of Aflatoxins

Quantitative determination of total aflatoxin was carried out using commercially available Veratox for Aflatoxin ELISA kit (Neogen Food Safety, Lansing, MI, United States) and measured on a Microplate Reader (Benchmark Plus; Bio-Rad, Laboratories Inc., Hercules, CA) at 600 nm (OD_600_).

### Six Fungal Strains From Korean Agricultural Culture Collection

The chemical structure of compound **1** produced by JCK-4087 was determined as CPA and then the fungal strain was identified as *A. flavus.* Because the fungal strain also produced aflatoxins, which are very strong carcinogenic mycotoxins, JCK-4087 cannot be used as a microbial nematicide. Therefore, we obtained six *P. commune* strains, known as CPA producers (Hermansen et al., 1984; Gqaleni et al., 1996; Ostry et al., 2018), from the Korean Agricultural Culture Collection (KACC). The J2s mortality of culture filtrates and ethyl acetate extracts of the six strains was tested against *M. incognita* as described (Cayrol et al., 1989; Nguyen et al., 2018). In addition, the production of CPA in the PDB culture filtrates of the six strains was analyzed by HPLC as described (Pourhosseini et al., 2020). Based on the HPLC results and *in vitro* bioassay, *P. commune* KACC 45973 was selected and then CPA was also isolated from the culture filtrate of the fungal strain using the same method as described above.

### Mortality Assay

Compound **1** extracted from *P. commune* 45973 fungal strain was evaluated J2s of *M. incognita* as previously described (Cayrol et al., 1989; Nguyen et al., 2018). Compound **1** was dissolved in methanol at a concentration of 30 mg mL^–1^, and its toxicity was tested against J2s of *M. arenaria, M. hapla*, and *M. incognita*. 1% Methanol was used as a negative control. J2s mortality was evaluated after 3 days of treatment and then calculated according to the following formula (Schneider and Orelli, 1947):


$$\begin{array}{c} \mathrm{Mortality}\left(\%\right)\\ =\frac{\begin{array}{c} \mathrm{Mortality}\mathrm{percent}\mathrm{of}\mathrm{treatment}-\mathrm{mortality}\mathrm{percent}\;\\ \mathrm{of}\mathrm{untreated}\mathrm{control}\; \end{array}}{100-\mathrm{mortality}\mathrm{percent}\mathrm{of}\mathrm{untreated}\mathrm{control}} \end{array}$$

The experiment was conducted with six replicates per treatment and was performed twice.

### Hatching Assay

Compound **1** dissolved in methanol was employed to immerse approximately 50 egg suspensions of the mixed-development stage in a 96-well tissue plate (Becton, Dickinson and Company, Franklin Lakes, NJ) at concentrations of 0, 5, 25, and 50 μg mL^–1^. The plates were sealed with parafilm to prevent evaporation and were kept in a humid chamber at 26 ± 2°C. The number of eggs and J2s of the three *Meloidogyne* spp. were counted at 0 (D_0_), 3 (D_3_), 7 (D_7_), 15 (D_15_), 21 (D_21_), and 28 (D_28_) days after treatment (Leica DM IL LED; Leica Microsystems CMS GmbH, Wetzlar, Germany). All the experiments were repeated twice with six replicates. The following formula was used to calculate the cumulative percent of egg hatch (Wu et al., 2014):


$$\mathrm{Cumulative}\mathrm{percent}\mathrm{of}\mathrm{egg}\mathrm{hatch}\left(\%\right)=\frac{\text{J}2sDx-\mathrm{J2s}\text{Do}}{\mathrm{EggDo}}\times 100$$

where Dx = x days after the start of the assay.

### Plackett–Burman Design

PBD was used to determine nutritional and environmental variables affecting the production of compound **1** (Plackett and Burman, 1946). The total number of experiments is n+1, where n is the number of variables. With 14 medium components (independent variables) and 20 experimental runs represented in two levels, the design matrix was used to evaluate independent factors that affected compound **1** production, as shown in Table 1. On PBD design, five dummy variables (D1–D5) did not affect the data analysis used to estimate experimental error (No, 2013; Phukon et al., 2020). Fourteen different independent variables were evaluated at two levels of high and low [denoted by (+1) and (−1, respectively; Table 1]. All the trials were carried out in triplicate, and the average concentration of compound **1** determined from the peak areas in HPLC chromatogram was considered the response variable, depending on the first–order Plackett–Burman model:


Y=βo+ΣβiXi

where Y is the concentration of compound **1** (the response or dependent variable), βo is the model intercept, βi is the linear coefficient, and Xi is the level of the independent variable.

**TABLE 1:** Coded and actual values of the medium used to produce cyclopiazonic acid by *Penicillium commune* KACC 45973 using Plackett–Burman design.

Symbol code	Variables	Units	Code values	
			− 1	1
A	NaNO_3_	g L^–1^	0	8
B	Tryptone	g L^–1^	0	8
C	Yeast extract	g L^–1^	0	8
D	Glucose	g L^–1^	0	90
E	Starch	g L^–1^	0	90
F	MgSO_4_.7H_2_O	g L^–1^	0.05	1
G	KCL	g L^–1^	0.05	1
H	FeSO_4_.7H_2_O	g L^–1^	0.001	0.02
J	K_2_HPO_4_	g L^–1^	1	2
K	PDB	g L^–1^	0	24
L	pH		5	8
M	Agitation speed	rpm	0	150
N	Incubation time	Days	14	21
O	Inoculum size	Plugs	5	10

### Response Surface Methodology

The optimal levels of the significant variables and the interactions of these variables during the production of compound **1** were analyzed by the CCD (Plackett and Burman, 1946; Lim et al., 2019). CCD was used to analyze three factors (NaNO_3_, tryptone, and yeast extract) at five levels [very low, low, intermediate, high, and very high as coded by numbers (−1.68), (−1), (0), (1), and (1.68), respectively]. The experiment was performed in triplicates. The average concentration of compound **1** from the HPLC analysis was considered the value of the response or dependent variable. For predicting the optimal point, the relationship between independent variables and the response or dependent variable was fitted in the quadratic polynomial of the second-order model:


Y=βo+ΣβiXi+ΣβiiXi+2ΣβijXiXj

where Y is the predicted response, βo is the regression coefficient, βi is the linear coefficient, βii is the quadratic coefficient, βij is the interaction coefficient, and Xi is the levels of independent variables. The interaction and quadratic terms are denoted by the *Xi*^2^ and *XiXj*.

### Disease Control Efficacy of the Wettable-Powder Type Formulation

The wettable-powder type formulation of the ethyl acetate extract of *P. commune* KACC 45973 (Pc45973–WP20) was prepared as previously described (Kim et al., 2018). The ethyl acetate extract was included in the formulation at a level of 20%. The disease control efficacy of Pc45973–WP20 was evaluated against tomato RKN disease caused by *M. incognita* using pot experiments. Susceptible tomato cv. Seokwang seeds were planted in nursery soil (Bunong horticulture nursery soil, Bunong, Korea) and maintained at 25 ± 2°C and 77 ± 5% RH for 4 weeks. Pc45973–WP20 was applied at 250, 500, and 1,000–fold dilutions by soil drench (20 mL per pot) twice (1 day before and 6 days after inoculation). A total of 1,500 J2s of *M. incognita* were inoculated into four-leaf stage tomato plants in a 9.5 cm diameter plastic pot containing a pasteurized nursery soil: sand (1:1, v/v) mixture. Sunchungtan^®^ containing 30% fosthiazate (SL; Farm Hannong Co., Seoul, Republic of Korea) was used as a positive control and applied twice at 4,000-fold dilutions. Distilled water was used as a negative control. The treated plants were arranged in a completely randomized design in the greenhouse at 28–33°C. Their roots were washed with tap water to remove adhered soil particles 6 weeks after the first application (Nguyen et al., 2018). Plant growth parameters, such as plant height and fresh weight of shoot and root, were recorded. Gall index (GI) was used based on a 0–5 galling scale, where 0 = 0–10%, 1 = 11–20%, 2 = 21–50%, 3 = 51–80%, 4 = 81–90%, and 5 = 91–100% root galls (Barker et al., 1985). Eggs and J2s were extracted from the root system and soil and counted under an optical microscope (Leica DM IL LED). Control values of gall index were calculated using the following equation (Yeon et al., 2019; Rajasekharan et al., 2020):


$$\begin{array}{c} \mathrm{Control}\mathrm{value}\left(\%\right)\\ =\frac{\begin{array}{c} (\text{galling}\mathrm{index}\mathrm{of}\mathrm{untreated}\mathrm{control}\;\\ -\mathrm{galling}\mathrm{index}\mathrm{of}\mathrm{treatment})\; \end{array}}{\text{galling}\mathrm{index}\mathrm{of}\mathrm{untreated}\mathrm{control}}\times 100 \end{array}$$

Nematodes were collected from a 100 cm^3^ soil sample using the modified Baermann technique and were then counted (Hajihassani et al., 2019; Kalaiselvi et al., 2019). The experiment was repeated twice with four replications per treatment.

### Statistical Analysis

The repeated measure ANOVA with SAS University Edition (SAS Institute Inc., Cary, NC) was used to analyze cumulative eggs hatch bioassay. Probability levels of *P* ≤ 0.05 were considered statistically significant. The 50% effective concentration (EC_50_) values were calculated by dose-response curves using the non-linear regression function of GraphPad Prism software version 8.0 (GraphPad Software, Inc.); these values were used for determining paralysis activity. Minitab Statistical Software (version 19, Minitab Inc., United States) was used for optimizing the statistical experimental design and performing regression ANOVA. For *in vitro* experiments, the one-way analysis of variance (ANOVA) with Tukey’s test was used with SPSS version 23.0 (SPSS Inc., Chicago, IL, United States).

## Resutls

### Identification of Fungal Strain JCK-4078

The fungal strain JCK-4087 was identified as *A. flavus* through phylogenetic and BLAST analysis using *ITS*, *Bt2*, and *Cmd* (Supplementary Figure 1). The *ITS*, *Bt2*, and *Cmd* sequences of JCK-4087 were deposited in Genbank under the accession numbers MW786751, MW894649, and MW894650, respectively. The phylogenetic tree constructed using *Cmd* provided better resolution in identifying the strain JCK-4087 than that constructed using *ITS* and *Bt2*.

### Nematicidal Activity of Fungal Strains

Three days after treatment, the culture filtrate of *A. flavus* JCK-4087 showed killing effects against J2s of *M. incognita* with an EC_50_ value of 3.48% (Supplementary Figure 2). Aflatoxin was detected at a concentration of 151 ppb in the culture filtrate of *A. flavus* JCK-4087 (data not shown). Conversely, the culture filtrates of *P. commune* strains obtained from KACC exhibited weak nematicidal activity against J2s of *M. incognita* (Supplementary Figure 3A). However, the ethyl acetate extracts of the six *P. commune* strains caused J2s mortality with EC_50_ values ranging from 312.5 to 653.3 μg mL^–1^ (Supplementary Figure 3B).

### Isolation and Identification of a Nematicidal Metabolite

One nematicidal metabolite (compound **1**) was purified from the crude extract of *A. flavus* JCK-4087. HR-ESI-MS of compound **1** displayed [M + H]^+^ molecular ion peak at *m/z* 337.15198 in the positive ion mode and [M + H]^–^ molecular ion peak at *m/z* 335.13750 [M − H] ^–^ in the negative ion mode, indicating its molecular formula to be C_20_H_20_N_2_O_3_ (Supplementary Figure 4). The UV-visible absorption spectrum of the compound showed the UV maxima at 223 and 279 nm (data not shown). NMR data of compound **1** are summarized in Supplementary Table 1 based on the ^1^H- and ^13^C-NMR, COSY, HSQC, and HMBC spectra. All the instrumental data of compound **1** were identical to those of CPA (Holzapfel, 1968; Luk et al., 1977; Lin et al., 2009). Therefore, compound **1** was identified as CPA. Among the six *P. commune* strains obtained from KACC, KACC 45973 produced CPA at the highest level (10.5 μg mL^–1^) in the PDB medium (Supplementary Table 2). Therefore, this fungal strain was used to further study the isolation of CPA, optimization of the culture fermentation process, formulation, and *in vivo* bioassay.

### Mortality and Cumulative Percent of Egg Hatch of Compound 1 Against *Meloidogyne* spp.

The nematicidal activity of CPA was tested against J2s of three *Meloidogyne* species (*M. incognita, M. hapla*, and *M. arenaria*). Compound **1** was more effective at causing mortality of *M. incognita* than of *M. arenaria* and *M. hapla* (Table 2). After 3 days, the EC_50_ value of compound **1** against J2s of *M. incognita* was 4.5 μg mL^–1^. In comparison, the EC_50_ values of compound **1** against *M. arenaria* and *M. hapla* were 60.51 and 18.82 μg mL^–1^, respectively.

**TABLE 2:** EC_50_ values (μg mL^–1^) for cyclopiazonic acid-induced mortality of *Meloidogyne incognita, Meloidogyne hapla*, and *Meloidogyne areaniana* after 72 h of nematode immersion in test solution with respective SE and 95% CI values.

Nematode species	EC_50_ (μg mL^–1^)	SE	95% CI
*M. incognita*	4.50	0.012	4.472–4.538
*M. hapla*	18.82	3.70	11.42–33.10
*M. arenaria*	60.51	10.96	40.15–110.30

*EC_50_, 50% effective concentration; SE, standard error; CI, confidence interval.*

**FIGURE 1:**
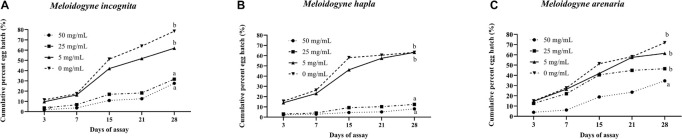
Effect of cyclopiazonic acid on cumulative percent of egg hatch of *Meloidogyne incognita* **(A)**, *Meloidogyne hapla* **(B)**, and *Meloidogyne arenaria* **(C)**. Different letters above bars indicate statistical significance based on Tukey–Kramer/Tukey’s HSD test (*P* ≤ 0.05). Each value represents the mean ± standard deviation of two experiments with three replicates.

Compound **1** also suppressed egg hatch of the three *Meloidogyne* species (Figure 1). The cumulative percentage of egg hatch of *M. incognita* and *M. hapla* at 28 days after exposure to compound **1** at 25 and 50 μg mL^–1^ was significantly lower than that of the untreated control of *M. incognita* and *M. hapla*, respectively. The egg hatch of *M. arenaria* was significantly reduced two times when exposed to only 50 μg mL^–1^ of compound **1**.

### Optimization by Plackett–Burman Design

PBD was used to identify the most significant variables affecting compound **1** production from *P. commune* strain. Compound **1** was produced in a wide range from 1.5 to 204.2 μg mL^–1^. Compound **1** production was the highest in run 11, followed by runs 14 and 17 (167.4 and 130.8 μg mL^–1^, respectively; Table 3).

**TABLE 3:** Twenty-trial Plackett–Burman experimental design for evaluation of independent variables with coded values along with the observed cyclopiazonic acid.

Run	A	B	C	D	E	F	G	H	J	K	L	M	N	O	D1	D2	D3	D4	D5	Actual value	
																				Area (mAU)	Conc.CPA (μg mL^–1^)
1	1	−1	−1	1	1	1	−1	1	−1	1	−1	1	−1	−1	−1	1	1	−1	1	87898.0	7.3
2	−1	1	−1	−1	−1	1	1	−1	1	−1	1	1	−1	−1	1	1	1	−1	1	61141.7	5.5
3	−1	−1	−1	1	1	1	1	−1	1	−1	−1	1	1	1	−1	1	−1	1	−1	0.0	1.5
4	1	−1	1	1	−1	1	1	1	1	−1	1	−1	1	−1	−1	−1	−1	−1	1	1434859.7	95.4
5	−1	−1	1	1	1	−1	1	−1	−1	1	1	−1	−1	−1	1	1	−1	1	1	213720.3	15.5
6	1	−1	−1	−1	−1	1	−1	1	1	1	1	−1	−1	1	1	1	−1	1	−1	117458.3	9.2
7	1	−1	1	−1	−1	−1	1	1	−1	−1	−1	1	1	−1	1	1	1	1	−1	75908.0	6.5
8	1	1	−1	1	1	−1	1	1	−1	−1	1	1	−1	1	1	−1	−1	−1	−1	54613.3	5.1
9	−1	−1	−1	−1	−1	−1	−1	−1	−1	−1	−1	−1	−1	−1	−1	−1	−1	−1	−1	32837.0	3.7
10	−1	−1	1	1	−1	1	−1	−1	−1	1	1	1	1	1	1	−1	1	−1	−1	0.0	1.5
11	1	1	1	−1	1	1	−1	−1	−1	−1	−1	−1	1	1	1	1	−1	−1	1	3099019.7	204.2
12	−1	1	1	−1	−1	1	1	1	−1	1	−1	1	−1	1	−1	−1	−1	1	1	100937.7	8.1
13	1	−1	1	−1	1	−1	−1	−1	1	−1	1	1	−1	1	−1	−1	1	1	1	1155138.3	77.1
14	1	1	1	1	−1	−1	1	−1	1	1	−1	−1	−1	1	−1	1	1	−1	−1	2536194.0	167.4
15	−1	1	1	−1	1	−1	−1	1	1	1	1	1	1	−1	−1	1	−1	−1	−1	248304.3	17.8
16	−1	1	1	1	1	1	−1	1	1	−1	−1	−1	−1	−1	1	−1	1	1	−1	0.0	1.5
17	1	1	−1	−1	1	1	1	−1	−1	1	1	−1	1	−1	−1	−1	1	1	−1	1976494.3	130.8
18	1	1	−1	1	−1	−1	−1	−1	1	1	−1	1	1	−1	1	−1	−1	1	1	232676.3	16.7
19	−1	−1	−1	−1	1	−1	1	1	1	1	−1	−1	1	1	1	−1	1	−1	1	1416.7	1.6
20	−1	1	−1	1	−1	−1	−1	1	−1	−1	1	−1	1	1	−1	1	1	1	1	681909.0	46.1

*mAU, milli-Absorbance Units; Conc.CPA, concentration of cyclopiazonic acid; D1–D5, dummy1-dummy5.*

**FIGURE 2:**
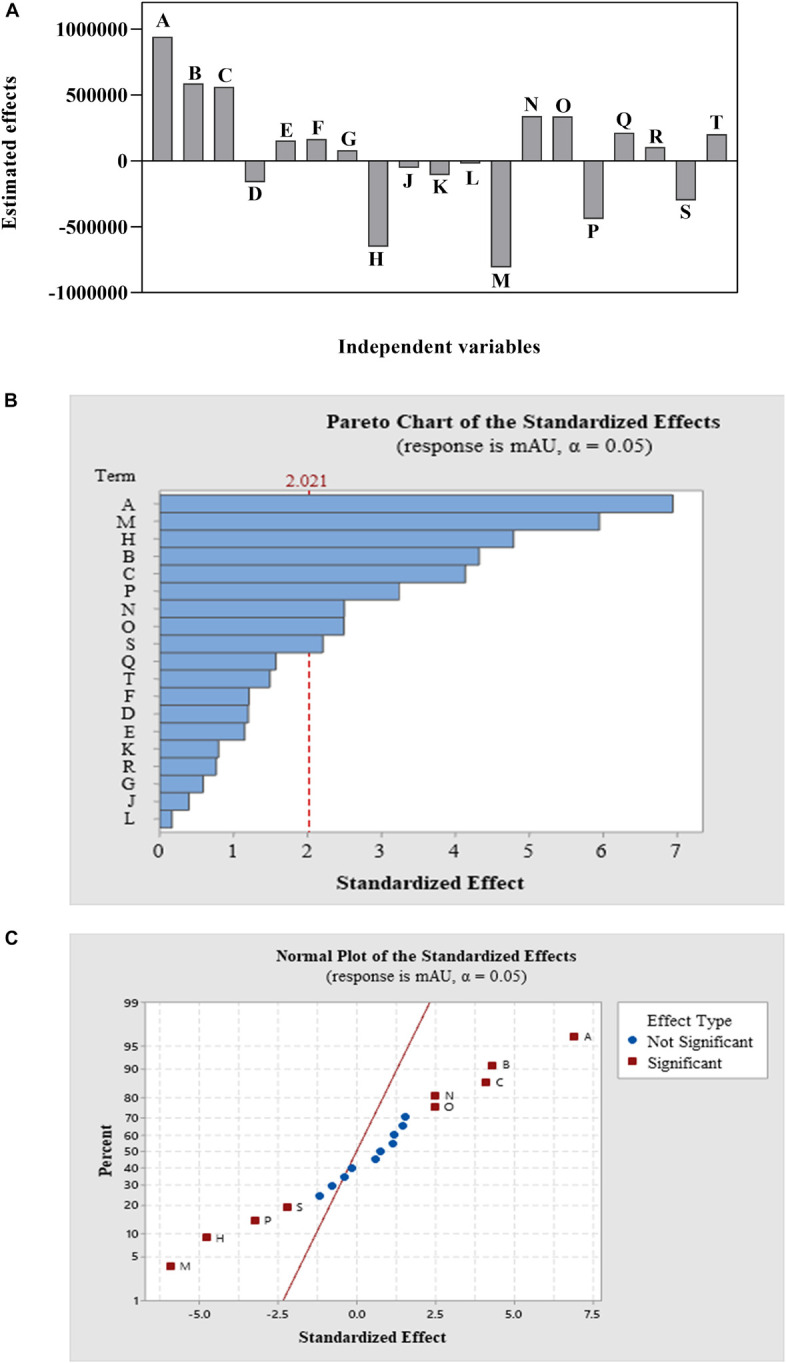
The main effects of the variables **(A)** and Pareto chart of the standardized effects of 14 variables design at a 95% confidence level **(B)** and the normal plot of the standardized effects of 14 variables design at a 95% confidence level **(C)** of a Plackett–Burman design for cyclopiazonic acid production by *Penicillium commune* KACC 45973.

ANOVA, as well as a sum of squares, mean squares, *F-value, t-value*, and *P-value*, were used to test the model’s adequacy. The statistical significance was determined using the *P*-value (probability value). Fisher’s statistical test (*F*-test) was also used for evaluating the statistical significance of the model. A model *F*-value of 9.49 and a *P*-value of 0.00 imply that the model is significant; there was merely a 0.0001% chance that a “Model *F-value*” this large could occur because of noise. Based on the ANOVA analysis, Table 4 indicates that the factor that contributed the most to compound **1** production is NaNO_3_, followed by agitation speed, FeSO_4_.7H_2_O, tryptone, and yeast extract in that order. The remaining variables did not contribute significantly to CPA production. Out of 14 factors affecting compound **1** production, only 3 factors—NaNO_3_ (A), tryptone (B), and yeast extract (C)—both positively and significantly caused an increase in CPA production (Figure 2 and Table 4). However, both agitation speed (M) and FeSO_4_.7H_2_O (H) exerted significant negative effects.

**TABLE 4:** Regression statistics and analysis of variance (ANOVA) for the experimental results of Plackett–Burman design used for cyclopiazonic acid production by *Penicillium commune* KACC 45973.

Source	Adj SS	DF	Adj MS	*F*-value	*p*-value	Contribution (%)
Model	5.00E+13	19	2.63E+12	9.49	0.00	
Linear	5.00E+13	19	2.63E+12	9.49	0.00	
A- NaNO_3_	1.33E+13	1	1.33E+13	48.09	0.00	21.82
B-Tryptone	5.17E+12	1	5.17E+12	18.65	0.00	8.46
C-Yeast extract	4.73E+12	1	4.73E+12	17.07	0.00	7.75
D-Glucose	3.97E+11	1	3.97E+11	1.43	0.24	0.65
E-Starch	3.66E+11	1	3.66E+11	1.32	0.26	0.60
F-MgSO_4_.7H_2_O	4.06E+11	1	4.06E+11	1.46	0.23	0.66
G-KCL	96010720299	1	96010720299	0.35	0.56	0.16
H-FeSO_4_.7H_2_O	6.35E+12	1	6.35E+12	22.88	0.00	10.38
J- K_2_HPO_4_	43118201686	1	43118201686	0.16	0.69	0.07
K-PDB	1.75E+11	1	1.75E+11	0.63	0.43	0.29
L-pH	7475950426	1	7475950426	0.03	0.87	0.01
M-Agitation speed	9.79E+12	1	9.79E+12	35.29	0.00	16.01
N-Incubation time	1.72E+12	1	1.72E+12	6.22	0.01	2.82
O-Inoculum size	1.72E+12	1	1.72E+12	6.19	0.01	2.81
P-Dummy 1	2.90E+12	1	2.90E+12	10.46	0.00	4.75
Q-Dummy 2	6.82E+11	1	6.82E+11	2.46	0.12	1.12
R-Dummy 3	1.63E+11	1	1.63E+11	0.59	0.44	0.27
S-Dummy 4	1.35E+12	1	1.35E+12	4.87	0.03	2.21
T-Dummy 5	6.16E+11	1	6.16E+11	2.22	0.14	1.01
Error	1.11E+13	40	2.77E+11			
Total	6.11E+13	59				

*R^2^, 81.85%; Adj R^2^, 73.23%; Pred R^2^, 59.16%; SS, a sum of a square; MS, mean square; DF, degree of freedom; F, Fisher’s function; P, level of significance.*

The value of the coefficient of determination (*R*^2^) was 81.85% of the variability in compound **1** production, indicating that only 18.15% of the total variances do not explain the independent factors. The high adjusted *R*^2^ (adj *R*^2^) value of 72.23% also points to the accuracy of the model. The predicted *R*^2^ (pred *R*^2^) value of 59.16% is in reasonable agreement with the adj *R*^2^ value of 81.73%. It proved that this model is good at predicting compound **1** production, matching between the observed values and the predicted response value.

The first-order polynomial Equation (3) was established based on the results of regression analysis and represents compound 1 production as a function of the independent variables that deserve the highest response:


$$\begin{array}{c} Y=605526+471500A+293603B+280882C-81339D\\ \;+78134E+82255F+40002G-325196H-26807J\\ \;-54016K-11162L-403865M+169532N+169142O\\ \;-219931P+106629Q+52084R-150120S+101345T\left(3\right) \end{array}$$

where Y is compound **1** production, and A, B, C, E, F, H, J, K, L, M, N, and O are NaNO_3_, tryptone, yeast extract, glucose, starch, MgSO_4._7H_2_O, KCL, FeSO_4_.7H_2_O, K_2_HPO_4_, PDB, pH, agitation speed, incubation time, and inoculum size, respectively.

NaNO_3_, tryptone, and yeast extract were chosen as the central points for further optimization using CCD based on the effect, coefficient, contribution, and *P*-value of each variable, as these factors had the most positive significant effects on CPA production.

### Optimization by Response Surface Methodology

In this study, a CCD was employed to optimize different levels of the three main factors (NaNO_3_, tryptone, and yeast extract) that affect compound **1** production from *P. commune* KACC 45973. Based on the experimental data obtained in Table 5, the concentrations of compound **1** ranged from 1.5 to 343.5 μg mL^–1^. The highest concentration of compound **1** produced from *P. commune* KACC 45973 (which represented the central point of CCD), was detected in run 20 (343.5 μg mL^–1^).

**TABLE 5:** Matrix of the central composite design and the corresponding experimental and predicted concentrations of cyclopiazonic acid produced by *Penicillium commune* KACC 45973.

StdOrder	Run	Variables			Area (mAU)	
		A	B	C	Experimental	Predicted
1	1	−1	−1	−1	0.00	469312.00
6	2	1	−1	1	657723.33	1344140.00
10	3	1.68179	0	0	3272122.00	3477750.00
5	4	−1	−1	1	1221872.67	1352719.00
16	5	0	0	0	3315119.33	3307446.00
15	6	0	0	0	3516230.33	3307446.00
20	7	0	0	0	4343611.00	3307446.00
19	8	0	0	0	2572035.67	3307446.00
17	9	0	0	0	4053908.33	3307446.00
3	10	−1	1	−1	862777.33	166334.00
11	11	0	−1.6818	0	296348.67	−26798.40
13	12	0	0	−1.6818	0.00	660673.00
9	13	−1.6818	0	0	0.00	−25874.20
12	14	0	1.68179	0	4334571.00	4671898.00
18	15	0	0	0	2211776.00	3307446.00
2	16	1	−1	−1	2349285.67	1599046.00
4	17	1	1	−1	4482329.00	4341456.00
7	18	−1	1	1	3691986.33	4198043.00
14	19	0	0	1.68179	4483061.00	386568.00
8	20	1	1	1	5229002.33	4984819.00
**Variables**	**Code symbol**	**Code values**				
		−1.68	−1	0	1	1.68
NaNO_3_	A	0	2	5	8	11
Tryptone	B	0	2	5	8	11
Yeast extract	C	0	2	5	8	11

*StdOrder, standard order; mAU, milli-Absorbance Units.*

The *R*^2^-value of 71.07% indicated that the three independent factors predicted 71.07% of the total variance in the dependent variable (compound **1** production) and that the model could not explain 28.93% of the total variance. In the quadratic model, the adj *R*^2^ (62.39%) and pred *R*^2^ (51.89%) values were found with an insignificant lack of fit (*P* > 0.05). All *R*^2^, adj *R*^2^, and pred *R*^2^ analyses indicated a good agreement between the experimental and predicted compound **1** and implicated that the analytical model fitted for stimulation of compound **1** production by *P. commune* KACC 45973.

The effects of NaNO_3_, tryptone, and yeast extract on the CPA production were analyzed based on the predicted response for producing compound **1** from *P. commune* KACC 45973 that can be expressed using a second-order polynomial model with coded symbols (A–N), as listed in Supplementary Table 3.


$$\begin{array}{c} Y=3307446+1041636A+1396933B+944201C\\ \;+198839AB-847086AC+224567BC\\ \;-{559149A}^{2}-{348214B}^{2}-{374352C}^{2} \end{array}$$

**FIGURE 3:**
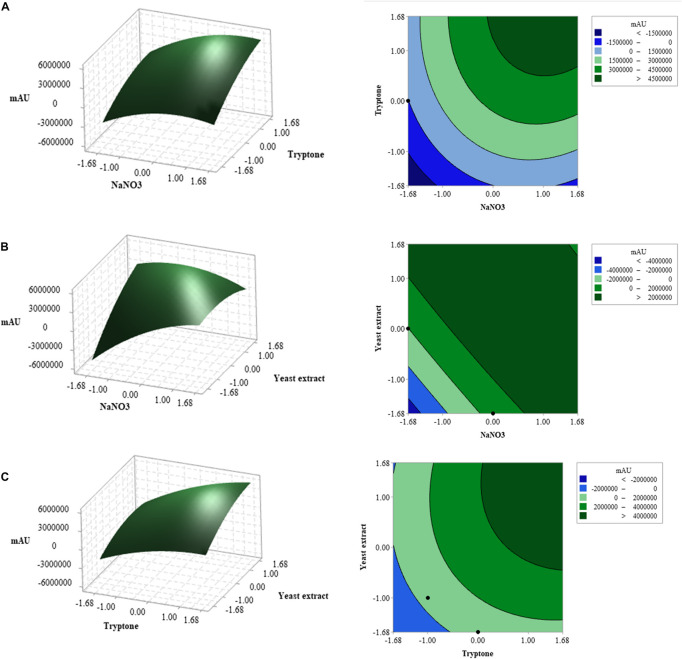
Three-dimensional (3D) response surface plots and two-dimensional (2D) contour plots of three factors affecting cyclopiazonic acid production by *Penicillium commune* KACC 45973. When the interaction of any two factors was plotted, the other factor was set at the central point: **(A)** tryptone and NaNO_3_, **(B)** yeast extract and NaNO_3_, and **(C)** tryptone and yeast extract.

The three-dimensional (3D) response surface and two-dimensional (2D) contour plots were subsequently used to graphically depict the interaction between the three variables. These plots displayed the combined effect of NaNO_3_ and tryptone on compound **1** production; the yeast extract was fixed at the central point (5 g L^–1^) (Figure 3A). Based on the ANOVA analysis (Table 6), both NaNO_3_ and tryptone were determined as significant variables affecting CPA production (*p-value* ≤ 0.05). The highest concentration of compound **1** produced was detected when the values of tryptone and NaNO_3_ were in the range + 1 to + 1.68. However, the ANOVA results showed that the interaction coefficient between these two variables was not significant (*p*-value> 0.05) (Table 6).

**TABLE 6:** Analysis of variance (ANOVA) of the quadratic model for cyclopiazonic acid production by *Penicillium commune* KACC 45973.

Source	Adj SS	DF	Adj MS	*F*-value	*p*-value	Contribution (%)
Model	1.59E+14	9	1.77E+13	7.22	0.00	56.52
Linear	1.21E+14	3	4.2E+13	17.17	0.00	43.14
A	2.33E+13	1	3.77E+13	15.4	0.00	8.30
B	6.86E+13	1	7.6E+13	31.09	0.00	24.38
C	2.94E+13	1	3.47E+13	14.2	0.00	10.46
Square	1.9E+13	3	6.57E+12	2.69	0.06	6.75
A^2^	9.81E+12	1	1.32E+13	5.38	0.02	3.49
B^2^	3.72E+12	1	5.18E+12	2.12	0.15	1.32
C^2^	5.45E+12	1	5.98E+12	2.45	0.12	1.94
2-Way interaction	1.86E+13	3	6.21E+12	2.54	0.07	6.63
AB	2.22E+12	1	7.25E+11	0.3	0.59	0.79
AC	1.53E+13	1	1.32E+13	5.38	0.02	5.45
BC	1.11E+12	1	1.11E+12	0.45	0.50	0.40
Error	1.22E+14	50	2.44E+12			43.48
Lack-of-fit	9.63E+12	5	1.93E+12	0.77	0.58	3.43
Pure error	1.13E+14	45	2.5E+12			40.05
Total	2.81E+14	59				100.00

*R^2^, 71.07%; Adj R^2^, 62.39%; Pred R^2^, 51.89%.*

*DF, degree of freedom; F, Fisher’s function; P, level of significance.*

Meanwhile, the combined effects of NaNO_3_ and yeast extract on compound **1** production were elucidated in Figure 3B; the concentration of tryptone was kept at the central point (5 g L^–1^). Based on ANOVA results, both NaNO_3_ and yeast extract affected compound **1** production, and interaction between these variables was significant at *P*-values ≤ 0.05 (Table 6). The maximum response was observed when the NaNO_3_ level was near +1 and the yeast extract level was between +1 and +1.68. Figure 3C illustrates the 3D response surface and 2D contour plots of compound **1** production by *P. commune* KACC 45973 as a function of tryptone and yeast extract, where the level of NaNO_3_ was kept at the center point (5 g L^–1^). The highest production of compound **1** was achieved when the levels of both variables were set in the range from +1 to +1.68. Furthermore, ANOVA analysis revealed that the interaction between the two variables contributed significantly to compound **1** production (*P* ≤ 0.05; Table 6).

### Validation of the Models Under the Optimized Setting

Three verification experiments were performed under various optimized medium conditions to confirm the validity and accuracy of the model. The independent factors’ design matrix was used along with the experiment results and theoretical values to predict compound **1** production (Table 7). The experimentally determined values of maximum compound **1** production and high mortality induced by the culture filtrate from *P. commune* KACC 45973 agreed with the predicted values (Table 7 and Supplementary Table 4). The mortality percent caused by the three optimal media was significantly higher than that by the PDB medium, suggesting that the equations are accurate and reliable for predicting compound **1** production by *P. commune* KACC 45973. Based on the finding, combining the selected factors is the best strategy to optimize the response depicted at the beginning of the study. This model is reasonable to optimize the parameters to increase compound **1** production. The optimum culture conditions for the CPA production by *P. commune* 45973 were determined as follows; liquid medium = the combination of NaNO_3_ (5.04 g L^–1^), tryptone (11 g L^–1^), yeast extract (11 g L^–1^), starch (90 g L^–1^), MgSO_4_.7H_2_O (1 g L^–1^), KCl (1 g L^–1^), FeSO_4_.7H_2_O (0.01 g L^–1^), K_2_HPO_4_ (2 g L^–1^), PDB (24 g L^–1^); an incubation time = 21 days; pH = 5.0; inoculum = 10 pieces of 5 mm agar plugs containing mycelia.

**TABLE 7:** Model validation experiments for cyclopiazonic acid (CPA) production from *Penicillium commune* KACC 45973.

No.	NaNO_3_ (g L^–1^)	Tryptone (g L^–1^)	Yeast extract (g L^–1^)	CPA yield (μg mL^–1^)			
				Predicted		Experimental	
				mAU	Conc.	mAU	Conc.
Medium 1	5.04	11	11	5,837,215	383.26	5,809,916	381.48
Medium 2	9.1	11	9.45	5,222,246	343.05	5,254,508	345.16
Medium 3	11	11	0.92	4,873,244	320.22	4,677,774	307.44

### Efficacy of Pc45973–WP20 Against *Meloidogyne incognita* J2s in Planta

From the *in vitro* results, CPA showed the highest nematicidal activity against *M. incognita* when compared with that against the other two *Meloidogyne* species. Thus, we investigated the potential biological control activity of CPA against *M. incognita in planta*. The disease control efficacy of Pc45973–WP20 against *M. incognita* on tomato plants was tested. *M. incognita* population (J2s and eggs) and gall formation on the tomato plants treated with Pc45973–WP 20 were significantly reduced when compared with those on the untreated control in a dose-dependent manner (Table 8). The positive control Sunchungtan completely controlled the gall formation and nematode development in the treated tomato plants. Additionally, there was no significant difference in plant height and fresh root weight among treatments, except with Sunchungtan, which caused phytotoxic effects such as reduced plant growth.

**TABLE 8:** Effect of the wettable powder formulation of ethyl acetate layer of *Penicillium commune* KACC 45973 (Pc45973–WP20) on gall formation, nematode populations in roots and soils, and shoot and root growth of tomato plants infected with *Meloidogyne incognita.*

Treatments	Conc. (folds)	Nematode population (J2 and eggs)		GI	Control value (%) of gall	Plant		Root weight
						
		g^–1^ root	mL^–1^ soil			Height (cm)	Weight (g)	
**Pc45973–WP20**	250	431.26 ± 37.84^*c*^	15.02 ± 1.38^*b*^	2.00 ± 0.82^*c*^	57.89 ± 16.3^*c*^	36.50 ± 4.50^*b*^	24.85 ± 3.60^*c*^	6.94 ± 1.22^*b*^
	500	510.39 ± 53.84^*c*^	16.74 ± 2.37^*b*^	3.00 ± 0.82^*c*^	36.84 ± 16.3^*bc*^	41.30 ± 2.50^*b*^	24.64 ± 1.45^*c*^	7.02 ± 1.10^*b*^
	1,000	703.89 ± 63.72^*b*^	20.26 ± 4.17^*b*^	3.75 ± 0.5^*b*^	21.05 ± 10.00^*ab*^	40.00 ± 4.10^*b*^	24.67 ± 2.66^*c*^	6.65 ± 0.29^*b*^
**Sunchungtan**	4,000	0.00 ± 0.00^*d*^	5.51 ± 5.60^*c*^	0.00 ± 0.00^*d*^	100.00 ± 0.00^*d*^	13.80 ± 11.10^*a*^	3.29 ± 2.83^*a*^	1.93 ± 0.52^*a*^
**Untreated control**	−	1233.07 ± 36.47^*a*^	32.96 ± 4.23^*a*^	4.75 ± 0.50^*a*^	0.00 ± 0.00^*a*^	31.30 ± 2.50^*b*^	17.16 ± 3.68^*b*^	6.17 ± 0.19^*b*^

*Means with same letters are not significantly different (p < 0.05) according to Turkey’s test. Each value represents the mean ± standard deviation of four repeated values from two trials.*

## Discussion

Numerous researchers have focused on developing fungal and bacterial biocontrol agents to control nematodes in agriculture (Abd-Elgawad and Askary, 2018; Forghani and Hajihassani, 2020). Many studies on the two genera *Penicillium* and *Aspergillus* have been conducted to explore various nematicidal metabolites against RKNs (Kusano et al., 2000; Nakahara et al., 2004; Siddiqui and Akhtar, 2009; Murslain et al., 2014; Jang et al., 2016). At 300 mg L^–1^, three compounds isolated from *Penicillium bilaiae* have shown nematicidal activities of 77, 52, and 98% against *Pratylenchus penetrans*, respectively (Nakahara et al., 2004). Furthermore, peniprequinolone, peniprequinolone A and B, 3-methoxy-4-hydroxy-4-(4′-methoxy-4 (4′-methoxyphenyl)) quinolinone, and cyclopenin isolated from *Penicillium* cf. *simplicissimum* have displayed weak nematicidal activity against *P. penetrans* (Kusano et al., 2000). In our previous studies, kojic acid isolated from *Aspergillus oryzae* exhibited nematicidal activity against *M. incognita* (Kim et al., 2016), and oxalic acid produced by *Aspergillus niger* F22 showed 100% J2s mortality and 95% egg hatch inhibitory activity against *M. incognita* at 2 mmol L^–1^ (Jang et al., 2016). In our present study, the culture filtrate of *A. flavus* JCK-4087 showed strong nematicidal activity against *M. incognita*. Recently Naz et al. (2021), also reported that the culture filtrate of one *A. flavus* strain had both eggs hatching inhibitory activity and J2s mortality of *M. incoginta*. Compared to J2s mortality between the two *A. flavus* strains, JCK-4087 showed much stronger nematicidal activity than the *A. flavus* strain reported (Naz et al., 2021) with J2s mortality values of 100% at a concentration of 20% for the former and about 40% at a concentration of 25% for the latter.

Both *A. flavus* JCK-4087 and *P. commune* KACC 45973 were found to produce CPA as a nematicidal metabolite. CPA has been reported to be produced by many fungal strains belonging to the genera *Penicillium* and *Aspergillus* (Hermansen et al., 1984; Trucksess et al., 1987; Ostry et al., 2018). The metabolite is toxic at high concentrations (Purchase, 1971; Van Rensburg, 1984; Morrissey et al., 1985) and has antibacterial activity against methicillin-resistant *S. aureus* (MRSA), *B. subtilis*, and *S. aureus* (Shang et al., 2012; Hong et al., 2015). To the best of our knowledge, this is the first report of the effects of CPA on J2s mortality and egg hatch of three *Meloidogyne* species.

In this study, the medium optimized via the statistical optimization approach enhanced CPA production. The first-order model based on PBD has indicated that the most statistically significant variables influencing CPA production were NaNO_3_, tryptone, and yeast extract. The adj *R*^2^ (72.23%) value indicates that the model is reasonable compared to previous studies (Nelofer et al., 2012; El-Naggar et al., 2016). A previous study showed that NaNO_3_ caused trap formation and exhibited nematicidal activity in *A. oligiospora* (Liang et al., 2016). Among the nitrogen sources considered in the present study, NaNO_3_ had the highest contribution (21.82%) and exerted significant effects to enhance CPA production (Zarabi et al., 2012). CCD was used to determine the optimal levels of NaNO_3_, tryptone, and yeast extract as selected variables for increasing CPA production. Overall, the maximum CPA production obtained (381.48 mg L^–1^) by *P. commune* KACC 45973 is greater than that by *P. commune* (3.99 mg L^–1^) (Gqaleni et al., 1996) and *A. flavus* (6.256 mg L^–1^) (Gqaleni et al., 1997) in the previous studies. Both *P. commune* IMI87247 and *P. commune* NRRL891 strains have been reported to produce CPA on GMP agar medium but not in submerged medium (Hermansen et al., 1984). In our study, CPA was produced in a submerged medium by *P. commune* KACC 45973.

Additionally, Pc45973–WP20 effectively suppressed the development of tomato RKN disease caused by *M. incognita* in the pot experiment. Similarly previous studies, *Penicillum* and *Aspergillus* species have been reported as potential biocontrol agents against RKNs under greenhouse and field conditions (Siddiqui and Akhtar, 2009; Murslain et al., 2014; Jang et al., 2016). For instance, *Penicillum chrysogenum* reduced galls caused by *Meloidogyne javanica* and *M. incognita* on tomato and cucumber plants under greenhouse conditions (Gotlieb et al., 2003; Sikandar et al., 2019). Furthermore, the compound brefeldin isolated from *Penicillum brefeldianum* HS-1 showed a reduction in gall numbers up to 41.4% after 4 weeks of *M. incognita* inoculation (Miao et al., 2019).

CPA is a mycotoxin that is produced by *Aspergillus* and *Penicillium* (Le Bars, 1979; Trucksess et al., 1987; Lin et al., 2009; Ostry et al., 2018; Rao et al., 2021). Even though CPA has been detected in a number of food sources such as peanuts, wheat, sunflower, meat, milk, and cheese, few studies have reported CPA as a mycotoxin because of its benign toxicity and low levels (Urano et al., 1992; Dorner et al., 1994; Burdock and Flamm, 2000; Oliveira et al., 2006; Rao et al., 2021). In addition, CPA has not been considered chronically toxic, and it does not affect the immune system (Burdock and Flamm, 2000; King et al., 2011). Hence, CPA can be used as a biochemical biopesticide with the contingency of risk assessments before commercialization.

Chemical nematicides have been used as the most effective means to control RKNs. However, the long-term use of carbamate and organophosphorus nematicides has increased nematode resistance, resulting in reduced field efficacy (Ebone et al., 2019; Desaeger et al., 2020). This has led to the development of novel chemical nematicides with new action mechanisms. CPA is an ergoline alkaloid and indol-tetramic acid. It is a specific inhibitor of the Ca^2+^-ATPase of sarcoplasmic reticulum (SERCA ATPase) (Seidler et al., 1989; Plenge-Tellechea et al., 2001; Moncoq et al., 2007; Chang et al., 2009). It is selective in inhibiting SERCA ATPase because it does not affect plasma membrane Ca^2+^ pumps. CPA has a little different mode of action with other SERCA ATPase inhibitors; CPA depletes 1,4,5triphosphate-sensitive Ca^2+^ stores and does not inhibit L-type calcium-channel activity, but thapsigargin and 2,5-t-butyl hydroquinone do (Nelson et al., 1994). Even though we do not perform any experiment on the mode of action of CPA against *M. incognita*, CPA may show nematicidal activity against J2s and egg hatching of *M. incognita* by inhibiting SERCA ATPase because it has an essential role in the muscle contraction-relaxation cycle. On the other hand, the V-ATPase inhibitor pyocyanin was also reported to show mortality against J2 of *M. incognita*, whereas ouabain, an inhibitor of the plasma membrane Na^+^/K^+^ ATPase, was ineffective (Caboni et al., 2013). This indicates that the nematicidal activity is quite different among ATPase inhibitors. To our knowledge, there is still no commercial nematicides that used CPA as the active ingredient, thus indicating that CPA can play an important role as a lead molecule to develop new chemical nematicides.

## Conclusion

In this study, CPA showed strong J2s mortality and egg hatching inhibitory activity against *Meloidogyne* spp. CPA production of *P. commune* KACC 45973 was enhanced by statistical methods using PBD and CCD. The optimized culture conditions resulted in 7.88-fold higher CPA yields than the basal medium conditions. Our findings indicate that CPA produced by *P. commune* KACC 45973 could be used directly as a biochemical nematicide or indirectly as a lead molecule of synthetic nematicides for controlling RKN diseases.

## Data Availability Statement

The data presented in the study are deposited in the GenBank repository, accession numbers MW786751, MW894649, and MW894650.

## Author Contributions

VN and J-CK conceived this study. VN, NY, YL, IH, and J-CK performed the experiments. VN, NY, HB, and J-CK analyzed the data. VN, HB, and J-CK wrote the manuscript. All authors approved the manuscript.

## Conflict of Interest

The authors declare that the research was conducted in the absence of any commercial or financial relationships that could be construed as a potential conflict of interest.

## Publisher’s Note

All claims expressed in this article are solely those of the authors and do not necessarily represent those of their affiliated organizations, or those of the publisher, the editors and the reviewers. Any product that may be evaluated in this article, or claim that may be made by its manufacturer, is not guaranteed or endorsed by the publisher.
