# Supplementary material for: Nematicidal Activity of Cyclopiazonic Acid Derived From Penicillium commune Against Root-Knot Nematodes and Optimization of the Culture Fermentation Process
Source: Front Microbiol. 2021 Nov 24;12:726504. doi: 10.3389/fmicb.2021.726504 (PMC8651706; doi:10.3389/fmicb.2021.726504)
Supplement: Supplementary file 1 [file Data_Sheet_1.docx]

Supplementary Material

# Supplementary Figures and Tables

## Supplementary Figures


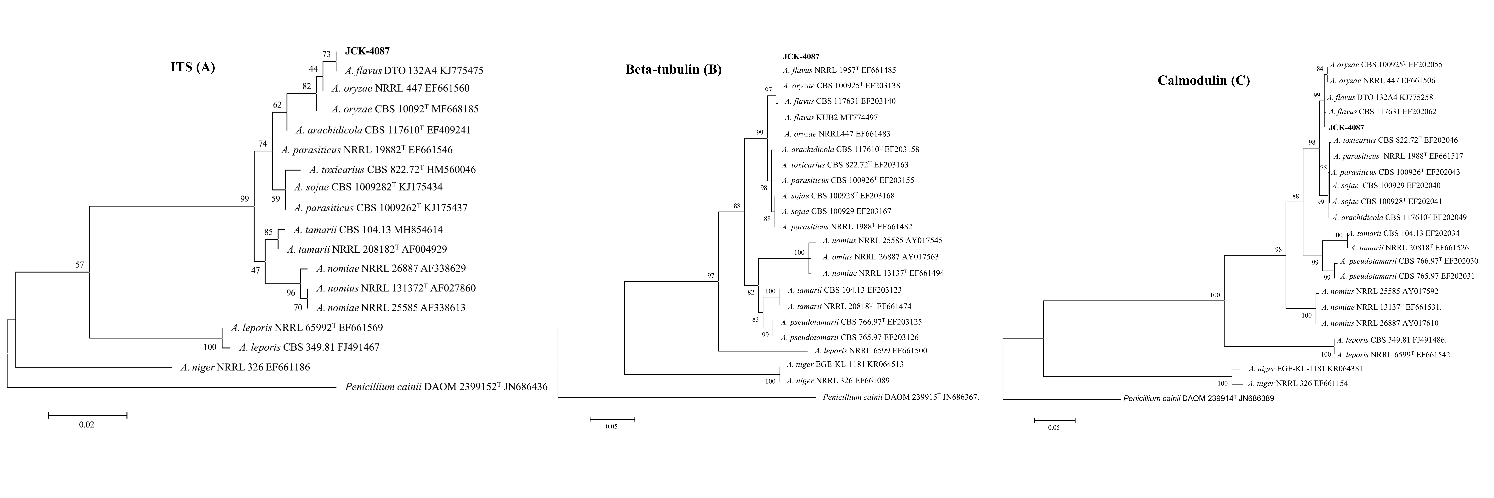


**Supplementary Figure 1.** Maximum likelihood phylogenetic tree reconstruction using the Tamura-Nei model based on the alignment of *ITS* (**A**), *beta-tubulin* (**B**), and *calmodulin* (**C**) gene sequences of JCK-4087. Bootstrap values (1000 trials) greater than 50% are displayed at the internal nodes. MEGA 6 software was used for aligning the sequences. T represents the type strains. The tree was rooted with *Penicillium cainii* DAOM 239914.

##
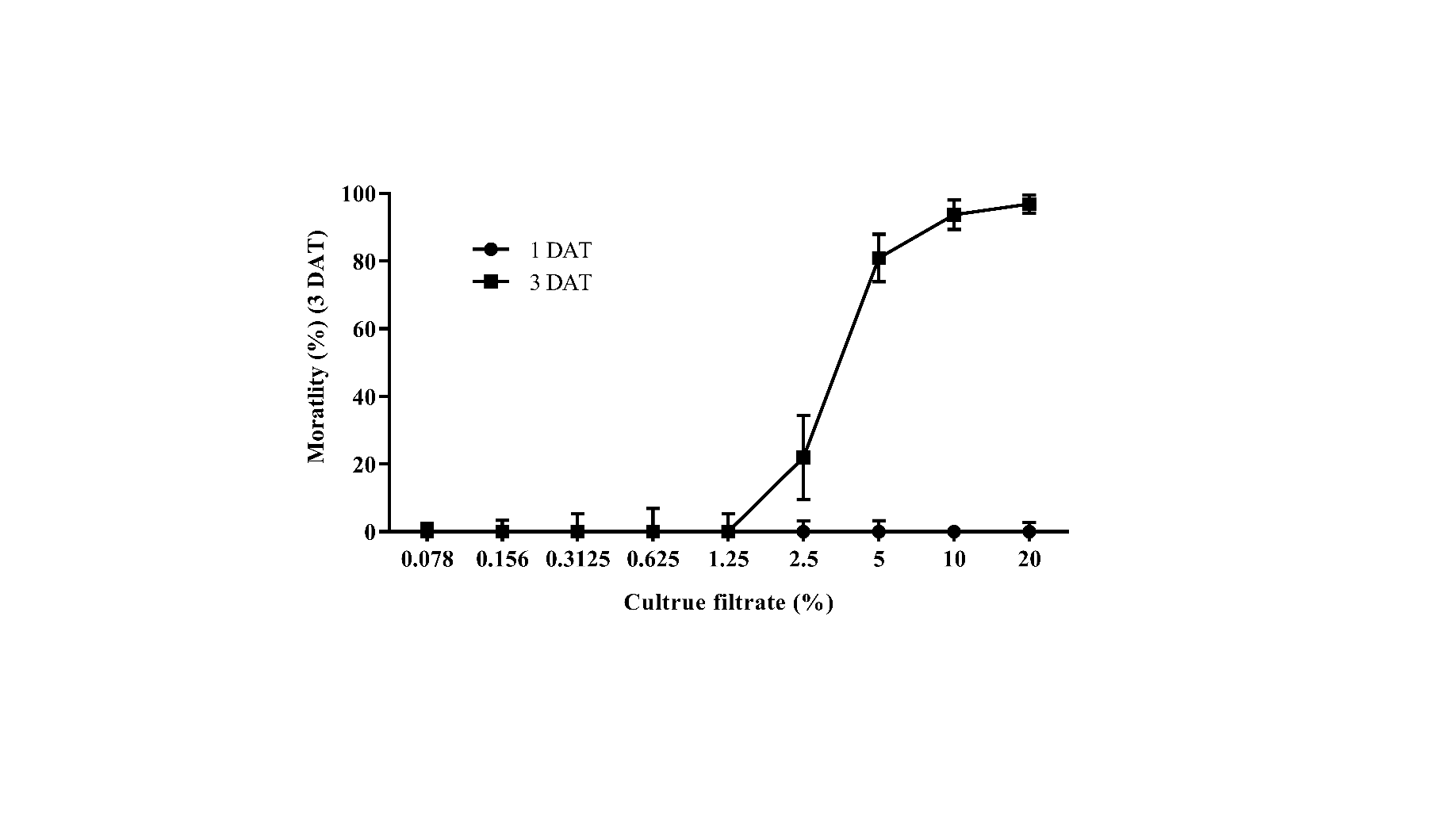


## Supplementary Figure 2. Nematicidal activity of the culture filtrate of JCK-4087 strain against the second stage juveniles of *Meloidogyne incognita* at one and three days after treatment. The fungus was cultured in potato dextrose broth medium at 25^o^C and 150 rpm for two weeks. Values are mean ± standard deviation (n=6).

**(B)**

**(A)**

**Supplementary Figure 3.** Nematicidal activity of the culture filtrate (**A**) and ethyl acetate layer (**B**) of *Penicillium commune* strains against the second stage juveniles of *Meloidogyne incognita* three days after treatment. The fungus was cultured in potato dextrose broth medium at 25^o^C and 150 rpm for two weeks. Values are mean ± standard deviation (n=6).


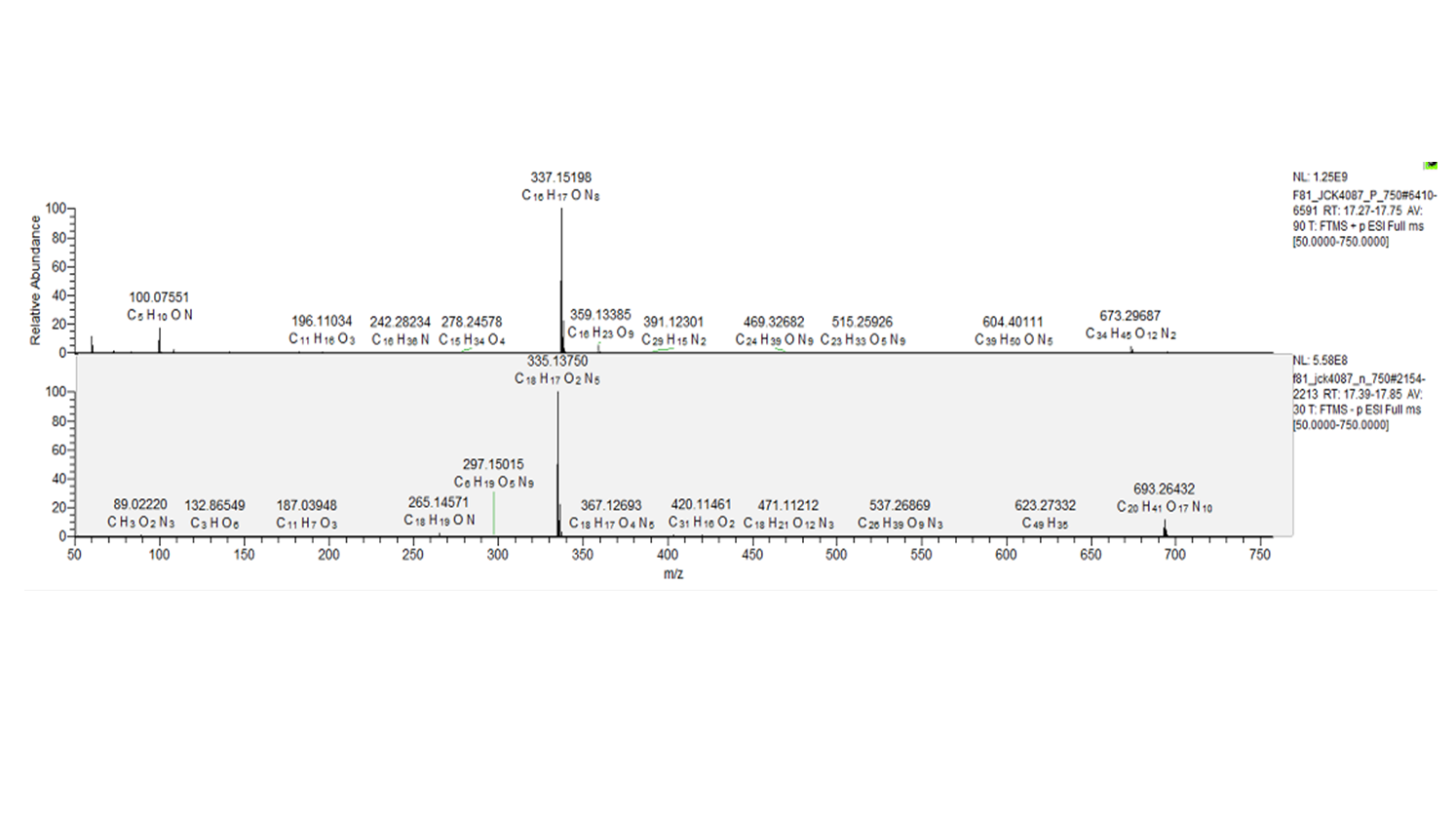


**(A)**

**(B)**

**Supplementary Figure 4.** LC-ESI-MS data of cyclopiazonic acid isolated from *Aspergillus flavus* JCK-4087 with positive (**A**) and negative ion mode (**B**).

**Supplementary Table 1** | ^1^H and ^13^C NMR (500 and 125 MHz, methanol-d4, TMS, δ, ppm) data of cyclopiazonic acid isolated from *Aspergillus flavus* JCK-4087

| **No.** | **δ_C_** | **δ_H_ (J/Hz)** | **HMBC** |
| --- | --- | --- | --- |
| 2 | 123.43 | 7.03 (1 H, t) | C-13. C-17 |
| 3 | 111.32 |  |  |
| 4 | 38.3 | 3.54 (1 H, brs) | C-3 |
| 5 | 71.09 | 3.91 (1H, dd, J = 9, 9 ) | C-8 |
| 6 | 199.14 |  |  |
| 7 | 106.67 |  |  |
| 8 | 179.42 |  |  |
| (N) 9 |  |  |  |
| 10 | 63.77 |  |  |
| 11 | 55.31 | 2.5 (1H, s) |  |
| 12 | 27.91 | 3.03 (2H, m) | C-11.C-14, C-17, C-18 |
| 13 | 130.11 |  |  |
| 14 | 116.78 | 6.79 (1H,dd, J=5.5) | C-12, C-14 |
| 15 | 122.23 | 7.13 (1H, t, d,J =9.5) | C-14, C-17 |
| 16 | 109.51 | 7.13 (1H, t, d,J =9.5) | C-17 |
| 17 | 135.43 |  |  |
| 18 | 127.59 |  |  |
| 19 | 195.57 |  |  |
| 20 | 27.31 | 2.38 (3H, m) | C-7, C-19 |
| 21 | 25.73 | 1.58 (3H, s) | C-10, C-11, C-22 |
| 22 | 26.69 | 1.63 (3H, m) | C-11, C-21 |

**Supplementary Table 2** | Production of cyclopiazonic acid of six fungal strains received from Korea Agricultural Cultrure Collection in potato dextrose broth medium incubated at 25°C and 150 rpm for two weeks

| **No.** | **Scientific name** | **KACC No.** | **Production of CPA**  **(µg mL^-1^)** |
| --- | --- | --- | --- |
| 1 | *Penicillium commune* | 45975 | 6.5 |
| 2 | *Penicillium commune* | 45974 | 6.2 |
| 3 | *Penicillium commune* | 45973 | 10.5 |
| 4 | *Penicillium commune* | 45972 | 2.6 |
| 5 | *Penicillium commune* | 45904 | 9.7 |
| 6 | *Penicillium commune* | 45404 | 8.7 |
| 7 | *Penicillium griseofulvum* | 44512 | 0 |

**Supplementary Table 3** | Regression statistic of CCD for optimization of cyclopiazonic acid production by *Penicillium commune* 45973

| **Term** | **Coef** | **SE Coef** | **95% CI Low** | **95% CI High** |
| --- | --- | --- | --- | --- |
| Constant | 3307446 | 366651 | 2571006 | 4043885 |
| NaNO_3_ | 1041636 | 265421 | 508521 | 1574750 |
| Tryptone | 1396933 | 250550 | 893689 | 1900178 |
| Yeast extracts | 944201 | 250550 | 440956 | 1447445 |
| NaNO3*NaNO3 | -559149 | 241074 | 1043361 | -74938 |
| Tryptone*Tryptone | -348214 | 239324 | -828911 | 132482 |
| Yeast extracts*Yeast extracts | -374352 | 239324 | -855049 | 106344 |
| NaNO3*Tryptone | 198839 | 365064 | -534414 | 932092 |
| NaNO3*Yeast extracts | -847086 | 365064 | -1580339 | -113833 |
| Tryptone*Yeast extracts | 224567 | 333033 | -444348 | 893483 |

**Supplementary Table 4** | EC_50_ value (%) with respective SE and 95% CI values of different optimal medium induced paralysis activity on the second stage juveniles of *Meloidogyne incognita* after 24, 48, and 72 hours of nematode immersion in test solution

| **Conc.  (%)** | **Medium 1 (hours)** | | | **Medium 2 (hours)** | | | **Medium 3 (hours)** | | | **PDB Medium (hours)** | | |
| --- | --- | --- | --- | --- | --- | --- | --- | --- | --- | --- | --- | --- |
|  | **24** | **48** | **72** | **24** | **48** | **72** | **24** | **48** | **72** | **24** | **48** | **72** |
| EC_50_ | 4.26 | 2.87 | 1.87 | 5.73 | 2.69 | 2.32 | 4.98 | 2.65 | 2.56 | 3007.00 | 702.00 | 340.00 |
| SE | 0.55 | 0.39 | 0.38 | 0.36 | 0.32 | 0.19 | 0.60 | 0.36 | 0.21 | 2542.00 | 1319.00 | 211.90 |
| 95% CI | 2.99 - 6.20 | 1.94 - 4.21 | 0.96 - 3.28 | 4.82 - 6.87 | 1.92 - 3.76 | 1.85 - 2.91 | 3.60 - 7.08 | 1.80 - 3.87 | 2.05 - 3.21 | 445.7 - 93416 | 59.60 - infinity | 99.27 - 4352 |
